# P-1879. Change in Healthcare Professional’s Identification, Counseling, and Adherence with Black Women for Long-Acting Cabotegravir (CAB LA) for PrEP Across Women’s Health, Primary Care, and Infectious Diseases Sites: Findings from the EBONI Study

**DOI:** 10.1093/ofid/ofae631.2040

**Published:** 2025-01-29

**Authors:** Dylan M Baker, Katherine L Nelson, Satish Mocherla, Yolanda Lawson, Alftan Dyson, Deanna Merrill, Lisa Petty, Peter Jeffery, Kenneth Sutton, Sara M Andrews, Samantha Chang, Kimberley Brown, Maggie Czarnogorski, Nanlesta Pilgrim

**Affiliations:** Emory University School of Medicine, Atlanta, Georgia; ViiV Healthcare, Durham, North Carolina; Legacy Community Health, Houston, Texas; Abounding Prosperity, Inc, Dallas, Texas; ViiV Healthcare, Durham, North Carolina; ViiV Healthcare, Durham, North Carolina; ViiV Healthcare, Durham, North Carolina; GSK, Brentford, England, United Kingdom; ViiV Healthcare, Durham, North Carolina; RTI International, Research Triangle Park, North Carolina; RTI International, Research Triangle Park, North Carolina; ViiV Healthcare, Durham, North Carolina; ViiV Healthcare, Durham, North Carolina; ViiV Healthcare, Durham, North Carolina

## Abstract

**Background:**

The CDC estimates that 400,000 Black women would benefit from PrEP but only 1% obtain a prescription for it in the US. Ensuring equitable access requires expanding delivery outside of infectious disease clinics and equipping healthcare professionals (HCPs) with strategies to support Black women’s PrEP use. EBONI is a phase 4 gender-concordant, implementation science trial evaluating the delivery of CAB LA, a new PrEP modality, to Black cisgender and transgender women in 15 US women’s health, primary care, and infectious disease sites. We present changes in HCPs’ perceptions of identifying, counseling, and supporting CAB LA use in Black women after four months of study implementation.

**Figure:**
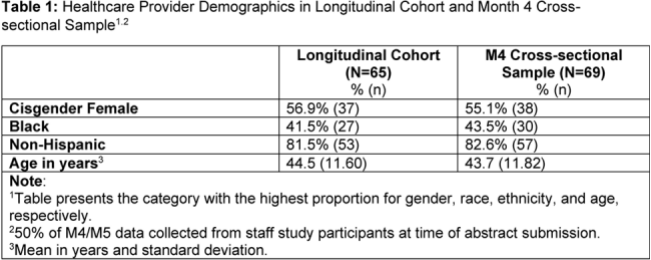

**Methods:**

Shifts from baseline (BL) to Month 4 (M4) in HCPs’ appropriateness, acceptability, feasibility and patient identification, counseling and adherence were assessed via surveys completed by a longitudinal sample of HCPs (N=65). At M4, a cross-sectional sample HCPs (N=69) evaluated usefulness of patient support tools. Descriptive statistics are reported.

**Figure:**
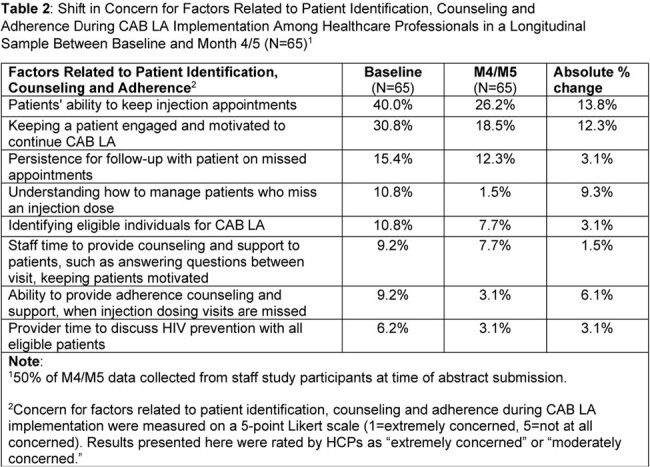

**Results:**

Table 1 presents demographics. HCPs reported high levels of appropriateness, acceptability, and feasibility of CAB LA (BL mean scale scores ≥4.6; M4 mean scale scores ≥4.3). Concerns decreased between BL and M4 for the perceived factors related to patient identification, counseling, and adherence (Table 2). HCPs reported feeling “extremely positive”/ “positive” about implementing CAB LA into care at BL (90.8%) and M4 (87.7%). At M4, of HCPs (50%) who completed the PrEP Education Training for Black Women training, most “completely agreed”/“agreed” the training would be useful to their practice (88%), helped them address patient HIV concerns effectively (84%), and improved their likelihood of providing PrEP (84%). Among HCPs (68.8%) that used a shared decision tool, most completely agreed/agreed the tool was easy to implement (82%) and supported conversations with Black women (82%).

**Conclusion:**

HCPs across women’s health, primary care, and infectious disease clinics maintained high appropriateness, acceptability, and feasibility after four months of integrating CAB LA into care. Effective tools exist to support HCPs in identifying, counseling, and supporting CAB LA use in Black women.

**Disclosures:**

Dylan M. Baker, MBBS, ViiV Healthcare: Grant/Research Support Katherine L. Nelson, PhD, MPH, GSK: Stocks/Bonds (Public Company)|ViiV Healthcare: Employee Yolanda Lawson, MD, Gilead: Honoraria|Gilead: Meeting and travel support|National Medical Association: Board Member|ViiV Healthcare: Advisor/Consultant|ViiV Healthcare: Grant/Research Support Alftan Dyson, PharmD, GSK: Stocks/Bonds (Public Company)|ViiV Healthcare: Employee Deanna Merrill, PharmD, MBA, AAHIVP, GSK: Stocks/Bonds (Public Company)|ViiV Healthcare: Employee Lisa Petty, MT(ASCP), GSK: Stocks/Bonds (Public Company)|ViiV Healthcare: Employee Peter Jeffery, BSc, PgDip, GSK: Complimentary Worker Kenneth Sutton, MA, GSK: Stocks/Bonds (Public Company)|ViiV Healthcare: Employee Kimberley Brown, PharmD, GSK: Stocks/Bonds (Public Company)|ViiV Healthcare: Employee Maggie Czarnogorski, MD MPH, GSK: Stocks/Bonds (Public Company)|ViiV Healthcare: Employee Nanlesta Pilgrim, PhD, GSK: Stocks/Bonds (Public Company)|ViiV Healthcare: Employee

